# Genome-based source attribution using a One Health Escherichia coli isolate collection from 2013 to 2023 in Scotland

**DOI:** 10.1099/mgen.0.001693

**Published:** 2026-04-20

**Authors:** Antonia Chalka, Louise Crozier, Adriana Vallejo-Trujillo, Alison Low, Sean McAteer, Vesa Qarkaxhija, Kate Templeton, Sue C. Tongue, Judith Evans, Geoff Foster, Catriona Webster, Thomas Evans, Charis A. Marwick, Ahmed Raza, Benjamin J. Parcell, Matthew T. G. Holden, Tom McNeilly, Stephen Fitzgerald, Mairi Mitchell, Nuno Silva, Emily Robertshaw-McFarlane8, Scott Hamilton, Beth Wells, Clare Hamilton, Eleanor Watson, David Finlay, Julie Bolland, John Redshaw, David Walker, Jane Heywood, Charlotte King, Craig Baker-Austin, Athina Papadopoulou, Andy Powell, Genever Morgan, Gavin K. Paterson, Jacqui McElhiney, David L. Gally

**Affiliations:** 1 The Roslin Institute and Royal (Dick) School of Veterinary Medicine, University of Edinburgh, Easter Bush Estate, Edinburgh, Scotland, EH25 9RG, UK; 2 Food Standards Scotland, Pilgrim House, Aberdeen, Scotland AB11 5RL, UK; 3 The New Royal Infirmary, NHS Lothian, Edinburgh, Scotland, EH1 3EG, UK; 4 Centre for Epidemiology & Planetary Health, School of Veterinary Medicine & Biosciences, SRUC, Rural and Veterinary Innovation Centre (RAVIC) Plot 9, Inverness Campus, Inverness, IV2 5NA, UK; 5 SRUC Veterinary Services, School of Veterinary Medicine & Biosciences, SRUC (Scotland’s Rural College), RAVIC Plot 9, Inverness Campus, Inverness, IV2 5NA, UK; 6 School of Infection and Immunity, Sir Graeme Davies Building, University of Glasgow, Glasgow, G12 8TA, UK; 7 Division of Population Health & Genomics, School of Medicine, Ninewells Campus, University of Dundee, Dundee, Scotland, UK; 8 Infection and Global Health Division, School of Medicine, University of St Andrews, North Haugh, St Andrews, Scotland, KY16 9TF, UK; 9 Moredun Research Institute, Pentland Science Park, Bush Loan, Penicuik, Midlothian, Scotland, EH26 0PZ, UK; 10 Scottish Environment Protection Agency (SEPA), Strathallan House, The Castle Business Park, Stirling, Scotland, FK9 4TZ, UK; 11 Centre for Environment, Fisheries and Aquaculture Science (CEFAS), Barrack Road, The Nothe, Weymouth, Dorset, DT4 8UB, UK; 12 Institute of Infection, Veterinary and Ecological Science, Leahurst Campus, University of Liverpool, Neston, CH64 7TE, UK

**Keywords:** *Escherichia coli*, machine learning, One Health, source attribution, wastewater

## Abstract

Random forest-based source attribution models were developed from a ‘One Health’ resource comprising 4,230 high-quality whole-genome assemblies from *Escherichia coli*. These were isolated from a wide range of sources, predominantly originating in Scotland, including wastewater, livestock, food and clinical infections of humans and dogs. Using these models, we derived a probabilistic assignment of *E. coli* isolates from food, shellfish and water samples to potential livestock and human sources of contamination. The incorporation of *E. coli* sequences from wastewater alongside those from human clinical infections enabled us to capture a wide diversity of human strains in our analyses. The sequence types (STs) of isolates from human bacteraemia and urinary tract infection (UTI) were compared with livestock and food isolates. While only 2.3% of the *E. coli* isolated from food samples in the study were from STs primarily associated with human bacteraemia and UTI, the models found a livestock signal associated with 15% of the human clinical isolates. In the food and private water samples, livestock-human co-attribution of *E. coli* isolates was common and consistent with routine human exposure to specific subsets of livestock *E. coli*, potentially a result of selection during food and water processing. Overall, this research demonstrates the potential value of including source attribution models in national surveillance programmes to understand the transmission of *E. coli* through the agri-food chain and support risk management to protect public health.

**Impact Statement**

This research examines the genetic composition of *Escherichia coli* isolated from many different sources including animals, humans, food, water and wastewater around Scotland. With the public resource generated, machine learning models were developed to allow the source of an *E. coli*, for example, one isolated from food or water, to be predicted from its genome sequence. We show that *E. coli* has genetic content associated with the originating host that allows source tracking using the developed models. Specifically, *E. coli* associated with human urinary tract infection and bloodstream infections are acquired predominately from human sources, although 15% of isolates exhibit livestock signals, especially pigs. Food, shellfish and water *E. coli* samples show low association with human clinical strains (~5%) but over a third of food isolates co-associate to both livestock and human sources, supporting food as key pathway to human colonization. The sequence and associated data provided is a valuable One Health resource, and the models generated can identify the animal or human source of an *E. coli* isolate. This can help with outbreak tracing and defining the sources of food or water contamination to develop appropriate interventions.

## Data Summary

The genomes and associated data can be viewed and interacted with at https://microreact.org/project/pathsafe1b-geninfo. The raw reads are available at https://enterobase.warwick.ac.uk/species/ecoli/search_strains?query=workspace:104476. Full scripts and packages available at GitHub: https://github.com/Antonia-Chalka/hostfinder. All data other than sequence data for the study are available in Tables S1–S14.

## Introduction

*Escherichia coli* is a ubiquitous commensal in livestock and humans and a key ‘sentinel’ bacterial species used for monitoring faecal contamination in water, assessing food hygiene and for qualifying and quantifying antimicrobial resistance (AMR) [1]. Therefore, *E. coli* can be part of a ‘one sample many analyses’ approach to One Health [2]. The ease and frequency with which *E. coli* can be isolated from many hosts and environments, including humans, livestock, water, soil and food vehicles [3,4], means that it is exploited to try and elucidate transmission routes between niches for both the microbe and the genes that it carries, especially those encoding AMR [5,7].

In humans, *E. coli* is the most common cause of human urinary tract infection (UTI) [8,9] and one of the most common causes of lethal bacterial sepsis from bloodstream infection (bacteraemia) [10,11]. In addition, Shiga toxin-encoding *E. coli* (STEC) continue to pose a threat to human health [12,13], and therefore, it is important to understand potential sources and routes of transmission of different *E. coli* subtypes to and from humans to protect public health.

The ability of *E. coli* to persist and thrive in many extremely different habitats is directly related to its genomic diversity. *E. coli* has a large pangenome of which ~25% of the gene content is core to the majority of strains [14,15]. This wide intra-species diversity makes source attribution for *E. coli* using whole-genome sequencing (WGS) challenging as large datasets are required to identify niche, host-related and/or geographically related signals.

Various types of genome-based signals (features) can be used to infer strain properties such as the originating source of an isolate. The application of machine learning (ML) to source attribution of *E. coli* is particularly promising, as it builds upon a growing understanding of the genomic basis of host adaptation while leveraging the predictive power of modern computational methods. *E. coli* exhibits distinct genomic signatures associated with different hosts and ecological niches, providing the biological foundation necessary for accurate source prediction [16]. ML offers a framework to capture these complex patterns, moving beyond traditional statistical approaches by prioritizing predictive accuracy, which is complementary to statistical inference that is crucial for extracting actionable insights from large genomic datasets [17].

In the last decade, ML has been applied to bacterial genome data to predict phenotypes such as AMR [18,21], metabolic pathways [22], disease severity [23,24] and phage infectivity [25] and to attribute the source animal [26,30] or geography [31,32] of an isolate. Studies on related pathogens, such as *Campylobacter* and *Listeria,* have already shown the power of this approach, using ML for source attribution while simultaneously exploring the dearth of approaches available [33,35]. For *E. coli*, these methods have been refined to achieve high specificity in identifying host sources [36].

Accurate source attribution helps to define the potential sources of pathogens, thereby supporting the investigation of outbreaks of human illness or contamination events, e.g. to ascertain the source (human, wild birds, canine or agricultural) of *E. coli* in water, which is currently of particular public interest in the UK. The availability of microbial WGS data from statutory and other clinical, environmental and food monitoring and surveillance programmes also provides a valuable source of information to support government strategy for tackling AMR [37].

The potential to use One Health genomics for bacterial foodborne pathogens resulted in a UK Government investment of over 20 million pounds in the PATHogen Surveillance in Agriculture, Food and the Environment (PATH-SAFE) programme [38]. Starting in 2021, this programme was led by the Food Standards Agency (FSA) and included pilot projects and infrastructure development for enabling the sharing of bacterial sequence datasets and allied metadata for public health benefit, as with GenomeTrakr in the USA [39]. Food Standards Scotland (FSS) commissioned a pilot project within PATH-SAFE to assess how source attribution modelling could be used within existing surveillance programmes to support understanding of *E. coli* transmission in Scotland. This manuscript reports on (1) the ‘One Health’ *E. coli* dataset established from ongoing sampling networks from across the country and (2) the generation of ML source attribution models applied to *E. coli* isolated from human clinical infections, food, shellfish and water.

## Methods

### Isolate collection and WGS

Isolates were collected from multiple laboratories in order to sample *E. coli* genomic diversity across Scotland including those involved in regulatory monitoring and food surveillance programmes commissioned by FSS, as well as those generated by research projects. Sources included livestock (poultry, ovine, bovine and swine), wild animals (deer and geese), companion animals (canine UTI), food (human and raw pet), humans (UTI and bacteraemia), the environment, drinking and recreational waters, wastewater and shellfish, primarily as samplers of bacteria in their local marine environments, with a view to capturing genetic diversity of *E. coli* across Scotland. All WGS was carried out by MicrobesNG (https://microbesng.com/). Either *E. coli* isolates or purified DNA were submitted, and Illumina sequencing (paired reads) was carried out as described (https://microbesng.com/documents/methods/). The Nextera XT Library Prep Kit (Illumina, San Diego, USA) was used. Libraries were sequenced on an Illumina NovaSeq 6000 (Illumina, San Diego, USA) using a 250 bp paired-end protocol. Raw reads were uploaded to EnteroBase [40,41] (https://enterobase.warwick.ac.uk/species/ecoli/search_strains?query=workspace:104476).

**Fig. 1.**
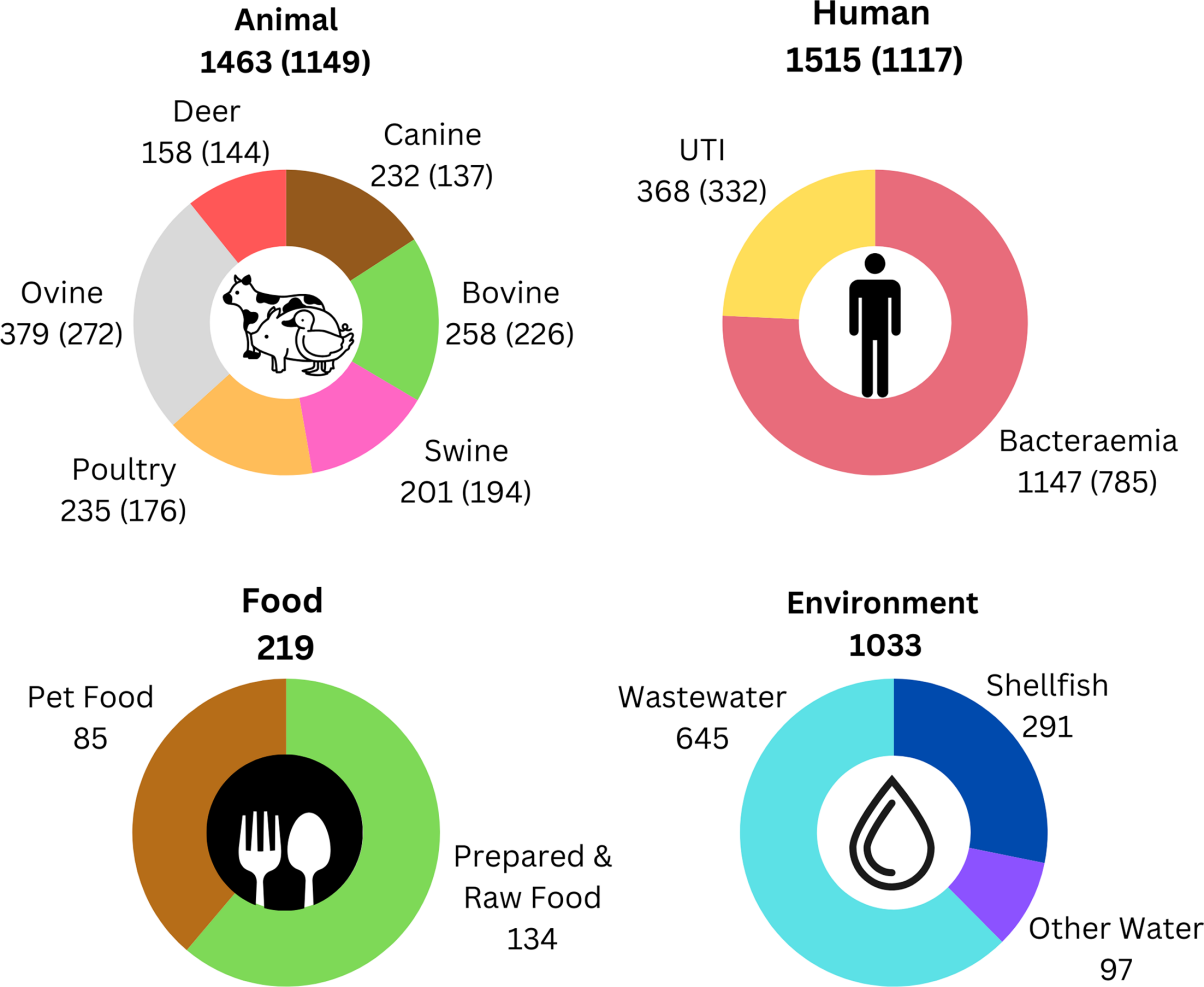
Breakdown of isolate numbers per host in the final 4230 dataset used for analysis. The numbers in parentheses represent the isolates used in the nonclonal dataset for model building. A summary of the isolate sources is provided in Table S2 and individual isolate metadata in Tables S1 and S3. The data can be visualized at https://microreact.org/project/pathsafe1b-geninfo.

Of 3,155 Scottish new *E. coli* isolates generated for the project, 3,142 were successfully sequenced and 2,960 assembled. These were combined with 1,354 existing assemblies (human and canine) that were generated through other projects (Fig. S1, available in the online Supplementary Material). For analysis, 4,230 assemblies passing EnteroBase QC and with <500 contigs were retained. Information on the sources of the isolates is provided in Fig. 1 (Results section) with complete details in Table S1 and a summary in Table S2. Imaging and sub-analysis of the data can be carried out at https://microreact.org/project/pathsafe1b-geninfo (we have not been easily able to remove two duplicated isolates so starting number is 4232 on the Microreact website).

### Sequence data analysis

The workflow for gathering high-quality assemblies, metadata, genome annotation and extraction of features to build and implement ML models was adapted from the methods presented in a previous study [26] and Fig. S2. High-quality assemblies were annotated with prokka:1.14.5 [42], with a nucleotide FASTA file of reviewed *E. coli* proteins from UniProt [43] inputted via the ‘trusted protein file’ option. panaroo:1.5.2 [44], run with options --remove-invalid-genes --merge_paralogs --clean-mode moderate, was used for pan-genome extraction.

For phylogenetic analysis, mlst:2.23.0 (Seemann T, mlst – Github https://github.com/tseemann/mlst) was used to define the sequence type (ST). This uses the PubMLST website (https://pubmlst.org/) [45]. ClermontTyping:24.02 [46] was used for phylogroup identification, and snippy:4.6.0 [47] was used to extract core SNPs with strain EC958 as the reference genome [48] with phylogenetic trees built with gubbins:3.2.1 [49] and iqtree 2.0.3 [50], as well as a binary pangenome presence/absence dendrogram. iTOL was used for tree visualization. SNP distances were calculated using snp-dists:0.8.2 [51] to identify epidemiologically linked isolates; those differing by <10 SNPs from the same host were classified as clonal. Of 2,978 high-quality human and animal assemblies, 2,266 nonclonal isolates were used for model training and testing (Table S3).

Predictions for both core SNP and pangenome trees were used to generate phylogenetic-prediction models based on 1–5 closest neighbours (Tables S4–S7). The most commonly occurring host was selected as the prediction. In the case of a tie, the host with the shortest average branch length was selected.

### Model generation

Models were created using the python scientific stack (python:3.11.9 [52], scikit-learn:1.5.1 [53], pandas:2.1.4, numpy:1.26.3 and pickleshare:0.7.5). Random forest was selected as the primary algorithm based on its strong performance in preliminary comparisons with alternative supervised algorithms (including support vector machines and gradient boosting) conducted during early stages of model development on a subset of data, consistent with findings from similar source tracking applications [26,30]. Random forest offers several advantages for this application: capacity to model non-linear feature interactions, resistance to overfitting with high-dimensional data and interpretability through feature importance metrics. The presence–absence matrix of pangenome gene clusters served as input features; as this represents a complete pangenome, no missing genomic data were present as all features were encoded as binary indicators (0/1) for each gene cluster.

Binomial classifiers were built for each host and the train/test split was stratified based on host, to ensure comparable ratios. Model performance was evaluated using Cohen’s kappa to account for chance agreement. Feature selection was performed using recursive feature elimination, and models were further refined with hyperparameter tuning. The threshold for prediction was adjusted based on PR curves, improving model efficiency compared to using default majority voting (i.e. a threshold of 0.5) [54].

Human attribution models included comparisons with ovine, bovine, swine, poultry and deer. Two sets of livestock models were created, one where only the livestock isolates were included (‘livestock-only’) and another that included human isolates and was labelled as ‘all’ to help remove *E. coli* from the wastewater set that might be from non-human sources. To build a ‘human–wastewater’ model aimed at capturing more of the diversity of *E. coli* excreted by humans, wastewater isolates with scores above threshold (defined by ROC/PR curves) in any ‘all’ animal model (ovine, bovine, swine, poultry, deer and canine) were excluded. Remaining wastewater isolates, presumed human-associated, were combined with clinical isolates.

To assess model generalizability, reduced human datasets excluded STs with <10 isolates and were sub-sampled to 700, 600, 500 and 400 isolates using StratifiedShuffleSplit [53] (Table S8). LGOCV-based on MLST clusters was used to evaluate model robustness to missing phylogenetic data.

Full prediction outputs, including ROC/PR adjustments, are in Table S9. To support public-facing interpretation, isolate attribution was visualized using ‘attribution profiles’, which stack confidence scores from each model, excluding those below threshold. This provides an overview of whether an isolate is attributed to one, multiple or no hosts. Visualizations were generated in R (R:s4.3.3, Tidyverse:2.0.0, readxl:1.4.3 and tidyverse:4.0.5), with full scripts and packages available at GitHub: https://github.com/Antonia-Chalka/hostfinder.

## Results

### Diversity of the *E. coli* dataset

From an initial collated set of 4,509 Scottish *E. coli* sequences, a final strain set of 4,230 high-quality assemblies was used in the study which passed quality control thresholds (Fig. 1, Tables S1–S3 and Fig. S1). This sequence set with metadata (host, time and, when available, geographical information and antimicrobial phenotyping) can be visualized and analysed at https://microreact.org/project/pathsafe1b-geninfo.

**Fig. 2.**
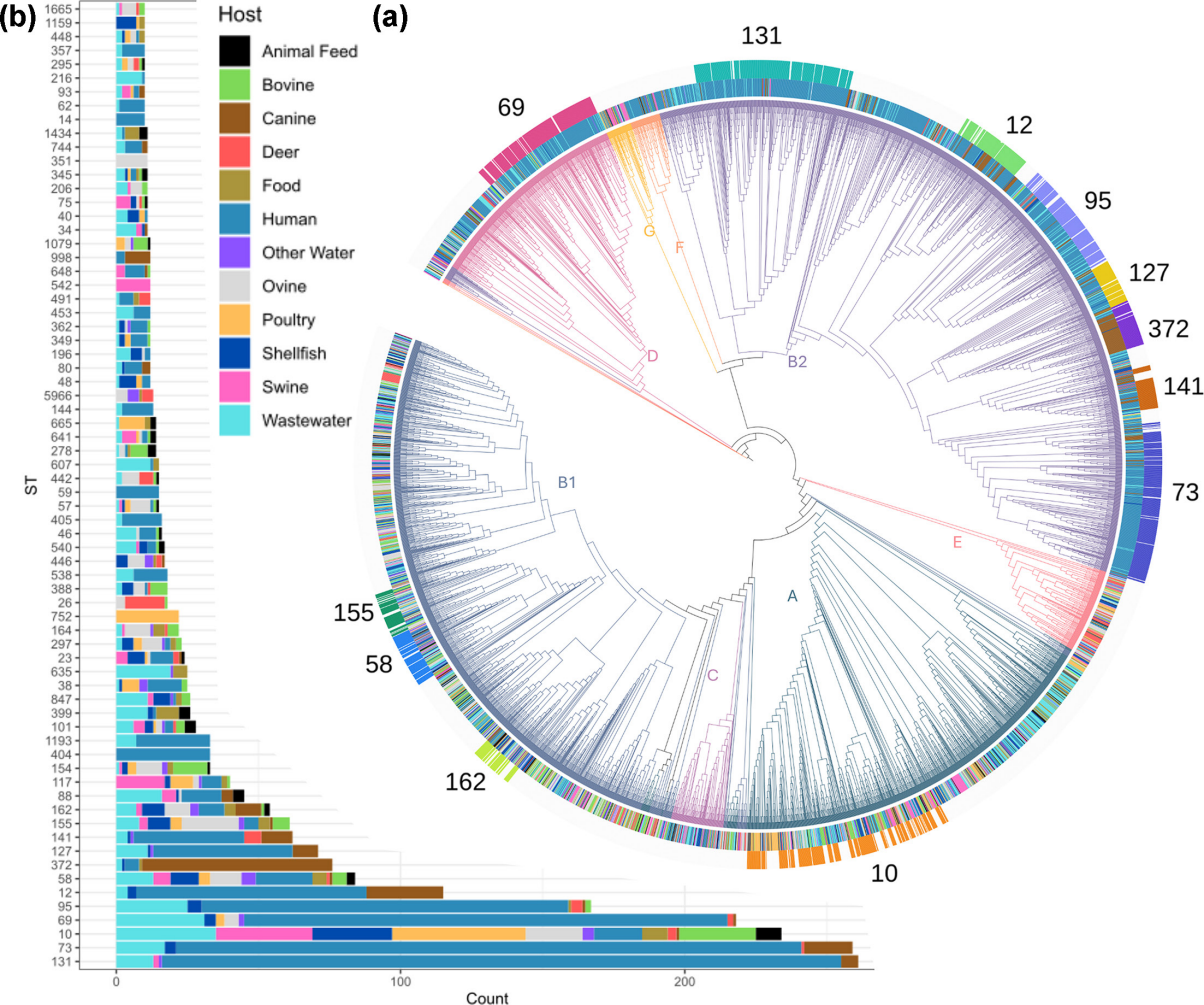
Phylogeny of the *E. coli* dataset. (**a**) Breakdown of the seven-gene MLST clusters with ten or more isolates by source. (**b**) Core SNP tree showing the phylogroup (coloured branches), source (inner coloured ring) and major seven-gene MLST clusters (outer coloured ring). A summary of the isolate sources is provided in Table S10 and individual isolate metadata in Table S11.

A whole-genome core SNP phylogeny (Fig. 2a) was generated for the 4,230 high-quality assemblies and is shown in relation to their isolation source with phylogroups (A–G) and predominant STs, according to the Achmann seven-gene MLST scheme. The relative distribution of isolates by source for every ST with over ten isolates is shown in Fig. 2(b). Phylogroup with host source is provided in Table S10 and ST (inc. subtype), phylogroup and serotype for each isolate in Table S11.

The dataset had 881 STs and host specialization of *E. coli* is apparent within many STs including 7 of the top 10 (STs 131, 73, 69, 95, 12, 127 and 141) that have predominately human ‘clinical’ isolates (UTI and bacteraemia), ST372 heavily populated with canine clinical strains, ST752 and ST665 with poultry isolates, ST542 for swine isolates and ST351 and ST57 for sheep isolates and deer isolates in ST26. It is also evident that isolates from the various sources are widely distributed and many STs contain isolates from multiple hosts, such as ST10 and ST58, making host assignment based on ST challenging.

Using only clinically derived human *E. coli* isolates would limit our ability to attribute food and water contamination to human sources using ML. We therefore incorporated wastewater isolates, as many will originate from human faecal matter and will capture some of the human-associated *E. coli* diversity absent from our clinical dataset. Wastewater isolates were distributed across phylogroups A (33%) and B1 (24%), consistent with commensal strains, with 25% in the more pathogenic B2/D phylogroups. The B2/D isolates clustered within the 172 STs dominated by the human clinical strains, supporting a primarily human source of the wastewater isolates. Most STs enriched from clinical isolates were also found in wastewater, though ST404, ST59 and ST14 were notable exceptions. As a proxy for relative virulence, the proportion of UTI and bacteraemia isolates associated with each main human clinical ST can be divided by their relative proportion in wastewater, i.e. an estimate of their general level in wastewater relative to their occurrence in UTI and bacteraemia infections. This ratio has ST131 as the most pathogenic with the following order across the most prevalent STs in the study: ST131 (7.85) > ST73 (5.52) > ST69 (2.33) > ST95 (2.19) > ST10 (0.21). Paired SNP distance analysis highlighted clusters of related isolates across the collection, especially within source groups but also between them, in particular bacteraemia-UTI cases (Tables S14 and S15). Specific examples are referred to in later sections.

Most food isolates, including pet food isolates, were evenly split across established commensal phylogroups A and B1. Only a small fraction of the *E. coli* isolated from human food (3/134, 2.2%) and raw pet food isolates (2/85, 2.4%) were found to cluster into the primary human pathogenic STs. These were defined as 172 STs out of the 881 STs in total, for which >50% of the isolates in the ST were from human clinical sources.

FSS undertakes a regulatory monitoring programme to assess the microbiological quality of shellfish growing sites in Scotland [55] which involves the isolation of *E. coli* from bivalve molluscs. A total of 291 *E. coli* isolates from 19 sites across Scotland were sequenced, and while the majority were in commensal phylogroups, 19 (6.5%) were in the majority human clinical STs.

### Source attribution models

Based on our published approach [26], random forest models were trained and tested with 2,266 nonclonal assemblies for binary classification, i.e. one host vs the rest, with gene cluster presence/absence as features. For ML predictions, different models were applied: the ‘livestock-only’ set of models for livestock prediction, a human clinical model and a ‘human–wastewater’ model in which the clinical human *E. coli* isolates were supplemented with a filtered set of wastewater isolates, as defined in Methods.

To benchmark the host attribution models, the accuracy of predictions was compared against other phylogenetic-based methods (Table 1). Specifically, two ‘nearest-neighbour’ phylogenetic models were built, one based on the core SNP tree (Fig. 2a) and the other on the pangenome dendrogram (Microreact). Models tested 1–5 nearest neighbours, with a single nearest neighbour yielding the best performance. Host prediction based on host ratios within each MLST cluster was also assessed.

**Table 1.** Accuracy metrics across different host attribution models To evaluate accuracy, both accuracy and kappa statistics were applied. For ML models, accuracy metrics for an unseen test set were generated, and the metrics used are based on PR-corrected thresholds. See Table S12 for a full list of the ML metrics and Table S13 for the list of isolates used as a test set for each ML model. Phylogenetic predictions were based on the nearest neighbour of a core SNP tree and a pangenome-based dendrogram. Finally, the accuracy of assigning the host to an isolate based on the majority host of the ST it belongs to was investigated.

Prediction method	Host model	Training accuracy (%)	Training kappa (%)	TEST accuracy (%)	TEST kappa (%)
**ML**	**Human**	99.7	99.5	97.5	95.0
	**Human wastewater**	99.6	99	90	79.2
	**Canine**	99.8	98.9	95.0	75.9
	**Deer**	100	100	98.2	91.5
	**Bovine**	98	93.9	85.7	59.6
	**Ovine**	99	97.4	88.5	69.8
	**Swine**	99.8	99.2	95.0	82.1
	**Poultry**	99.9	99.6	95.5	82.4
**Core SNP phylogeny** (**nearest neighbour**)	**Human**	87.7	75.0		
	**Canine**	93.3	73.0		
	**Deer**	93.2	64.8		
	**Bovine**	82.4	41.2		
	**Ovine**	81.7	52.8		
	**Swine**	90.5	55.8		
	**Poultry**	92.0	61.8		
**Pangenome dendrogram (nearest neighbour**)	**Human**	91.4	82.3		
	**Canine**	93.0	71.7		
	**Deer**	95.5	76.6		
	**Bovine**	84.6	48.0		
	**Ovine**	85.5	63.1		
	**Swine**	90.5	58.2		
	**Poultry**	92.6	64.4		
**MLST (plurality**)	**Human**	67.9			
	**Canine**	58.0			
	**Deer**	57.6			
	**Bovine**	46.9			
	**Ovine**	43.9			
	**Swine**	42.2			
	**Poultry**	41.5			

**Fig. 3.**
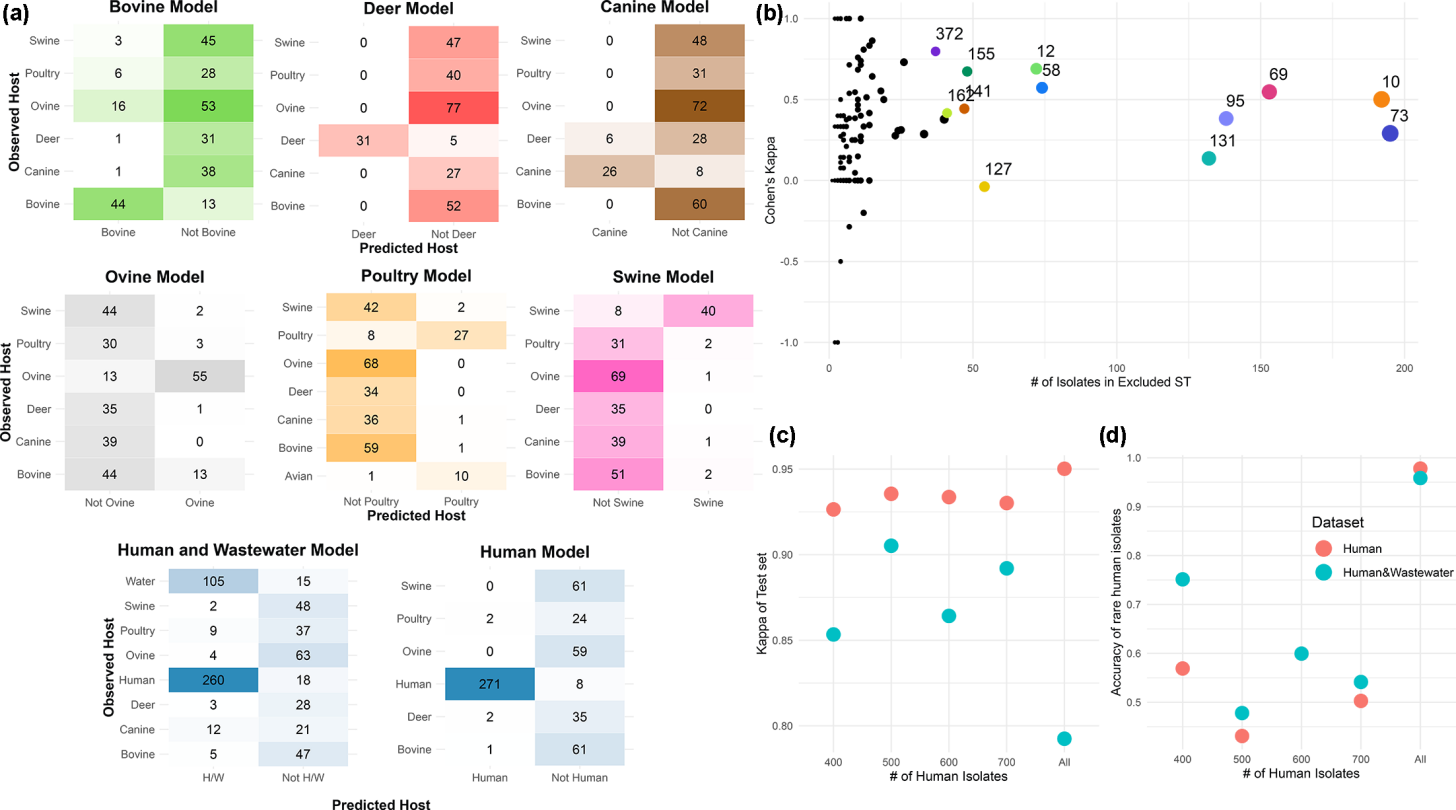
(**a**) Confusion matrices of the ML models’ test sets. As each model was trained as binary classifiers, the predicted hosts are limited to a host/not host classification. A list of test isolates for each model is provided in Table S13. (**b**) Leave-group-out model accuracy. For each model, an ST cluster was set as the test set, with the other ST isolates serving as the training/validation set. Major STs are coloured and labelled. (**c**) Performance of human (red) and human and wastewater models (blue) on complete vs reduced datasets. Model performance measured by the kappa of the test set. (**d**) Accuracy of the human isolates present in the STs excluded during dataset reduction. A list of which isolates made up each reduced model is provided in Table S8.

Phylogeny-based models achieved high raw accuracy (>80%) but showed lower performance on the kappa metric, which accounts for class imbalance, dropping to 40% for bovine, ~60% for most animal hosts and~75% for human (Table 1). Dendrogram-based predictions had higher kappa than the SNP tree but remained inferior to ML-based models. Model performance was further assessed using an independent test set excluded from training. While accuracy/kappa declined slightly, ML models remained robust: ~90% for human and deer, ~80% for swine and poultry, ~75% for wastewater and canine, 69% for ovine and 59% for bovine. Even the lowest-performing ML models outperformed phylogenetic approaches. The reduced accuracy for bovine and ovine models prompted closer examination of misclassifications. Most errors involved mutual misassignment between ovine and bovine isolates, indicating overlap in their genomic signatures (Fig. 3a). Livestock models were generally conservative, favouring false negatives over false positives, except in the ovine–bovine cross-over, a preference for non-assignment rather than misclassification. In contrast, the human model showed balanced false positive and negative rates (~0.02), while the wastewater–human model showed higher false positives (~0.15) than false negatives (0.07), indicating a tendency to over-assign isolates as human.

Known potential limitation of the gene-based source attribution models is a reliance on phylogeny for performance [26]. To test if this was present, models were generated using leave-group-out cross-validation based on the seven-gene MLST clusters. As expected, model accuracy varied per excluded clusters but generally led to models with reduced reliability confirming the importance of working within the test population structure (Fig. 3b).

One reason for model trends, as defined above, is the imbalanced dataset, in particular the higher number of human and human–wastewater isolates compared to those from other sources. To address this, these groups were sub-sampled while preserving diversity. Kappa values remained high, though accuracy declined for rare human STs excluded from training (Fig. 3c). For assigning isolates to a potential human source, the sub-sampled 600-isolate human–wastewater model was used, which balanced sample size with representative ST diversity (Fig. 3d).

### Source attribution of *E. coli* associated with human clinical disease, food, shellfish and water

#### UTI and bacteraemia isolates

Out of the 1,515 human clinical *E. coli* isolates, 228 (15.0%) were attributed to a livestock host model, often with a human co-attribution. Specifically, 150 (9.9%) isolates were attributed to swine and human, and 13 (0.9%) isolates were attributed to swine only. Twenty-one (1.4%) isolates were attributed to poultry and human and six (0.4%) to poultry only. Eight (0.5%) isolates were attributed to bovine and human and seven (0.5%) to bovine only. Four (0.3%) isolates were attributed to deer and human and one (0.1%) to deer only. Four (0.4%) isolates were attributed to ovine and human. A few isolates were attributed to three hosts, such as five (0.3%) isolates that were attributed to swine, poultry and human and two (0.1%) isolates that were attributed to bovine, swine and human. Some isolates were co-attributed to livestock only, such as five (0.3%) isolates that were attributed to swine and poultry and one (0.1%) isolate that was attributed to bovine and swine. So, while most human UTI and bloodstream infection strains appeared quite human-specific, a small subset has genetic content associated with livestock strains, particular pigs (*N*=176, 11.6%, when combining all associations above).

#### Food samples

**Fig. 4.**
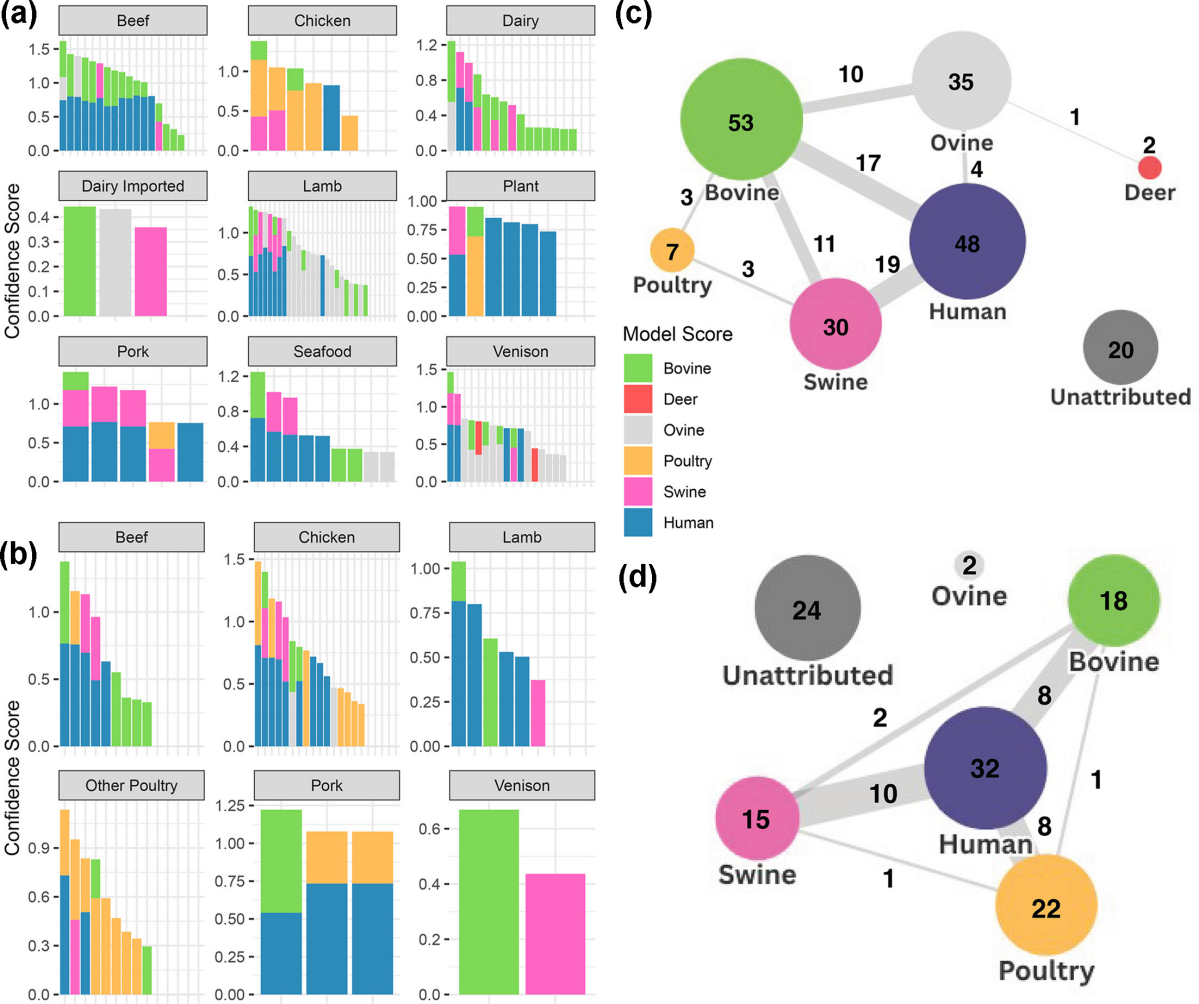
Food isolate attribution. The models used for human attribution are the human and wastewater reduced 600 models highlighted earlier (Fig. 3d). Only confidence scores above the threshold for attribution are shown for each sample. The full attributed food set is in Fig. S3. For bar graphs, each bar represents an individual isolate’s attribution profile. (a) Attribution of prepared and raw foods intended for human consumption. (**b**) Attribution of raw pet foods. (**c**) Network chart of human food isolate attribution. Each node (circles) represents the set of isolates assigned to a host or unassigned. The connecting lines represent the number of isolates that have multiple assignments across the two hosts. (**d**) Network chart of pet food isolate attribution. In both (c) and (d), the combined human and wastewater model was applied for the ‘human’ attribution.

In this study, 219 *E. coli* isolates were sequenced from various food sources, including raw meat, raw pet food, salads, dairy and ready-to-eat items. Attribution results (Fig. 4) are based on the livestock and reduced human–wastewater models. Among the 134 human food samples (Fig. 4a), 100 (74.6%) were confidently assigned to a livestock source. For raw pet food (*n*=85) (Fig. 4b), 40 (47.0%) of the confidently attributed isolates were found to match the expected production animal. Concordance was reasonable for beef, poultry and lamb products, despite the ovine–bovine overlap. In contrast, *E. coli* from venison is often misassigned to other species.

Of the 219 food-derived *E. coli* isolates (human and pet food combined), 94 (42.9%) were assigned only to livestock, while 80 (36.5%) showed significant human attribution, from which 58 (26.5%) co-assigned to a livestock source, while 22 (10.0%) were assigned only to the human–wastewater model. These were mostly non-pathogenic phylogroups and STs, although there were some exceptions: (1) an isolate from a chicken sandwich which assigned only to a human source from the models and this closely matched a clinical bloodstream isolate (3 SNPs apart) and (2) an isolate from a venison burger belonging to ST95, an important pathogen-associated ST. It is worth noting that 20% of the food isolates lacked any clear human or livestock attribution. The general relationships are summarized in Fig. 4(c) for human food and Fig. 4(d) for raw pet food with ≤5 SNP relationships summarized in Table S15 and detailed in Table S14.

#### Shellfish samples

**Fig. 5.**
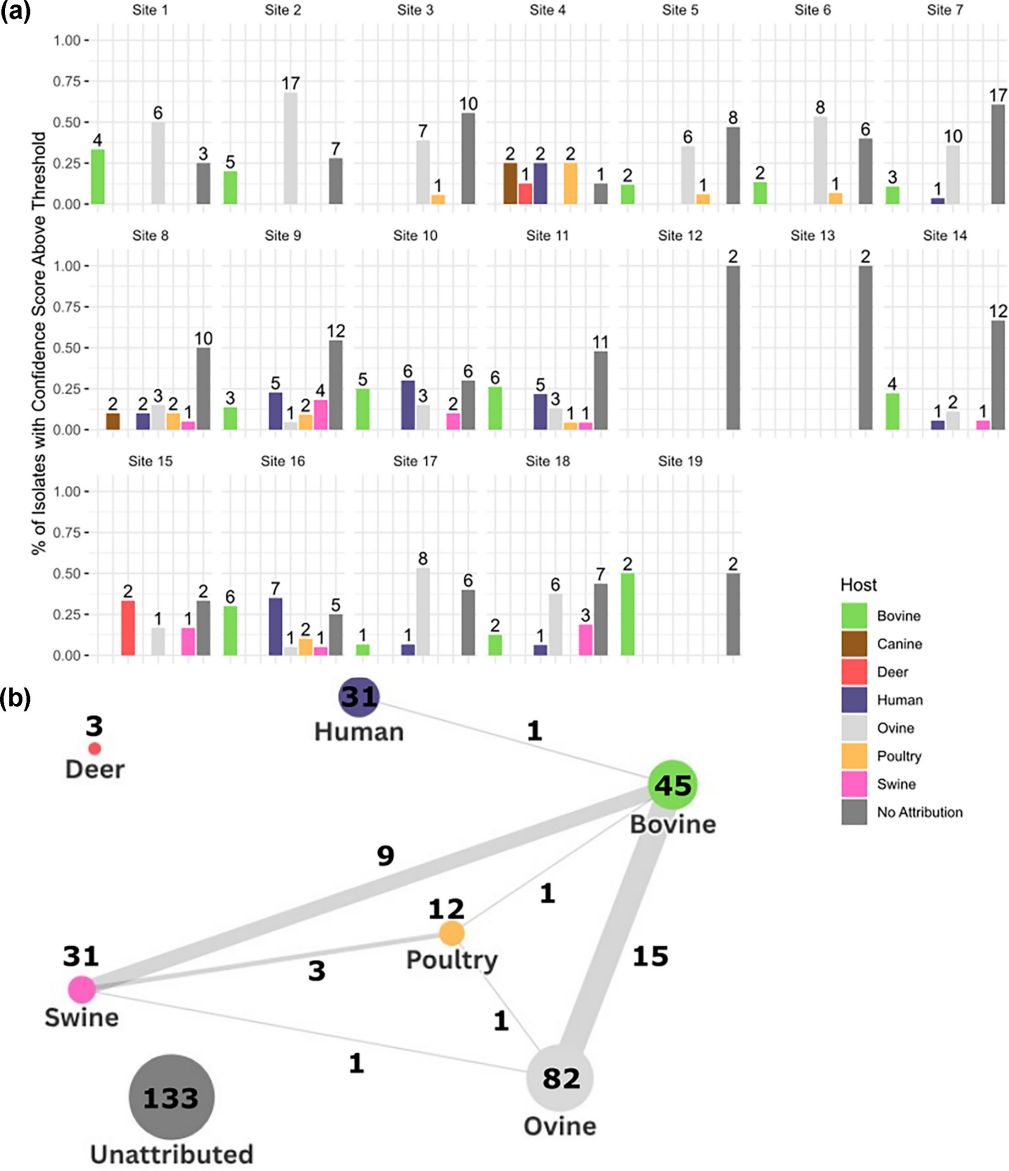
(**a**) Shellfish attribution per site. The human attribution is based on the selected human and wastewater model (see Methods). The percentage of isolates per site that were attributed to a host, as well as the actual number of isolates, is shown. Sites 12 and 13 only had a low number of isolates sampled (two at each site) with no assignment using the models. (**b**) Network chart of shellfish isolate attribution. Each node (circles) represents the set of shellfish isolates assigned to a host or unassigned. The connecting lines represent the number of isolates that have multiple assignments across the two hosts. I

The shellfish samples used in this study were collected from 19 different sites across Scotland and 291 sequences analysed using the source attribution models (Fig. 5).

The findings indicate that attribution varied markedly with respect to shellfish sampling location. Twelve of the 19 sites had a predominant ruminant attribution (ovine and bovine), which suggests that these sites are likely to be located in proximity to agricultural environments. Out of the 291 samples, 30 (10.3%) attributed exclusively to a human–wastewater source; for three sites, this was >20% of the samples, indicating that these sites may be at greater risk from wastewater discharge or direct human contamination. Other non-livestock sources of *E. coli*, such as canine (two sites), were detected (Fig. 5a). While many of the shellfish isolates belonged to rarer STs and will require a larger test collection set to ascribe the specific source of such isolates with higher confidence, there were 14 (4.8%) isolates belonging to the main human pathogenic lineage STs. It was notable that from the shellfish samples, there was virtually no co-attribution (one isolate only) of the human-assigned isolates with livestock sources. This contrasts with the high level of co-attribution demonstrated for human food and raw pet food isolates (Fig. 5b compared to Fig. 4c, d).

#### Water samples

Our analysis also included 97 *E. coli* isolates from other water samples. These originated mostly 73/97 (80.2%) from private water supplies, with the remainder comprising a mixture of environmental and recreational sources. Of the 73 private water supply isolates, 17 (23.2%) were assigned to human–wastewater only, 10 (13.7%) were assigned to both human and livestock and 56 (76.8%) were assigned only to livestock hosts. The network chart for private water isolates is provided as Fig. S4.

## Discussion

This study utilized national surveillance and monitoring programmes to compile a novel dataset of 4,230 *E. coli* genome assemblies from a range of One Health sources. A key application for the dataset was the development of host attribution models for *E. coli* using ML as a tool to support understanding of transmission pathways and potential sources of contamination for food and water samples. Building on prior work predicting isolate source and infection risk [26,29, 30], this study demonstrated that ML models, using differential gene features, outperformed phylogeny-based clustering in accuracy. As defined previously, model accuracy was heavily dependent on staying within the population diversity of the training set [26]. Specific models were developed, including one that combined sequence data from the human clinical isolates and a subset of wastewater isolates to capture a wider diversity of *E. coli* for attributing human/wastewater-based contamination.

A key limitation, and a focus of our model validation, is the issue of transferability. As defined previously [26], model accuracy was heavily dependent on staying within the population diversity of the training set. Our use of leave-one-group-out cross-validation (LGOCV), where entire source groups are held out during training, provides a better estimate of performance on unseen sources than random cross-validation. Models trained on the LGOCV approach had variable accuracy. Therefore, its accuracy in other geographical settings would be dependent on their phylogenetic makeup, as the composition of circulating strains and local ecological pressures may differ significantly. The predictive power of our models is inherently linked to the phylogeny represented in the training data; isolates with novel genetic backgrounds falling outside this learnt diversity would be assigned with low confidence. Therefore, while our models are robust for source attribution within the studied Scottish context, their application elsewhere would require careful validation with local isolate collections.

Assigning human origin to food and water isolates was challenging, but we propose that the inclusion of wastewater isolates should enhance the process. While excluding livestock isolates, based on our livestock models, from the combined human clinical–reduced wastewater model introduced some circularity, it likely enables representation of many human-associated *E. coli* not linked to disease. We deliberately termed the model ‘human and wastewater’ as we do not know the originating source of the wastewater isolates, although there is a high expectation that human excreta is the source of the majority of the *E. coli* present especially in major city outflows and this is, in part, supported by the ST analysis. The inclusion of the wastewater is valuable to capture the diverse, often non-clinical, human gut flora, but will also include some *E. coli* from other sources. This combined model can help identify isolates with a likely signature of human association, which could arise either from direct human faecal contamination or from contamination via wastewater.

Our study found that~25% of wastewater isolates belonged to the major clinical STs. This indicates their common carriage and faecal excretion by the general population. It was interesting to note that ST131 and ST73 showed the greatest disparity between their proportions in wastewater and proportional associations with clinical infections (UTI and bacteraemia). This ratio supports the idea that these particular STs have higher pathogenic potential rather than being present at higher overall frequencies in the human . More research is needed to compare levels and types of *E. coli* routinely excreted in faeces and urine from humans to understand how well wastewater samples provide a proxy for this.

While most human clinical (UTI and bacteraemia) isolates belonged to dominant human STs (131, 73, 69 and 95) [9], the source attribution models indicated that 16% may be linked to a livestock origin, with the highest attribution to pigs (11.6%). A study in the USA, based on mobile genetic elements [56], estimated that 8% of clinical *E. coli* (mainly UTI in their study) may have a meat-animal source. It is appreciated that the ML approach lacks the precision of SNP-based sub-clustering to identify, for example, host switching events and emergence of host specific sub-clusters. However, individual strains of *E. coli* can colonize multiple hosts, and a probabilistic approach, sometimes with multiple assignments, is a valid additional analysis to attribute sources of infections, even if the timescales for attribution are unclear.

A key finding was that many human and pet food isolates, even though attributing to a logical livestock source (for example, 70% of beef products were attributed to cattle) also had a significant human and wastewater co-attribution (80/219, 36.5% of the combined food isolates, Fig. 4c, d). This contrasts with the livestock attributed isolates from shellfish production sites which had very little human and wastewater co-attribution (1/291, 0.3%, Fig. 5b). The co-attribution of isolates from private water supplies was 10/73 (13.7%, Fig. S4). This would indicate that livestock *E. coli* isolated from food (and to a lesser extent private water) do not represent the wider diversity of *E. coli* excreted by livestock, with one explanation being that these isolates are specific subsets that survive food and water processing and then colonize humans as a result of routine exposure through consumption.

The prediction outputs retain continuous confidence scores rather than binary classifications, allowing for nuanced interpretation of attribution certainty. For surveillance applications, we provide the following guidance: low-confidence predictions (scores near thresholds) should be interpreted as normal in livestock models due to their conservative error rate, whereas human or human and wastewater predictions near the scores should be treated as more intermediate, in line with a more cautious approach. Isolates with scores above the threshold in multiple host models reflect ambiguous or mixed signals potentially indicating recent host switching or environmental mixing; a system of ranking predictions has not been developed in depth yet, although future work should implement a ranking based on the greatest distance from the threshold. Isolates with no scores above the threshold represent unassigned *E. coli*, the source of which cannot be confidently determined with current models.

In line with the above, 12% of the food isolates assigned uniquely (no other assignment) to a human source using the reduced human clinical and wastewater model, with a quarter of these (3% of food isolates) matched to major human clinical STs (UTI/bacteraemia). While the former (12%) could be examples of direct human contamination, we interpret them cautiously as indicators of a human faecal pathway, whether direct or via wastewater, that warrants further investigation in terms of hygiene practices.

The study also included samples from ranging ‘wild deer’, and there were individual examples of specific human origin pathogenic ST types (ST95, 73 and 69) being recovered from deer, potentially indicating their exposure to human contamination in the environment. In a similar way, shellfish *E. coli* levels are monitored as a signal of seawater contamination in the production area, and our models have then allowed source attribution of shellfish isolates from different cultivation sites around Scotland. Thirteen of the 19 sites had mainly ruminant-associated *E. coli* consistent with agricultural run-off into the shellfish production site. However, *E. coli* isolates from a few of the sites had low livestock but high relative human–wastewater attribution; in total, 10% of the shellfish isolates were assigned to only the human–wastewater model. There were several examples of shellfish isolates which were 0 and 1 SNP apart from those found in wastewater, indicating that the sites from which these samples were taken had been subject to contamination via wastewater. These findings highlight the potential of the source attribution models to provide information that could assist authorities and businesses in developing mitigation strategies which can manage the risks of contamination. Further validation of our attribution models against existing microbial source tracking methods would be useful to assess the value of integrating *E. coli* WGS into existing programmes for the monitoring and management of water that is intended for consumption, recreational and food production purposes. To further develop the models, as well as direct human gut isolate sequences, broader sampling is needed from sources like rodents, wild birds and natural environments including soil, water and plants. These will likely fill gaps where we currently have no assignment over our set thresholds.

## Conclusion

This study demonstrates the potential value of ‘multi-source’ isolates for the development of ML attribution models and how these models can be strengthened through the integration of wastewater isolates to increase the diversity of human associated strains available for analysis. Although it only provides a snapshot of *E. coli* diversity, the dataset signposts strain transfer across humans, animals, food and water. It also provides a valuable resource for interrogating AMR allele flow in a One Health network, and research is ongoing with this analysis. These attribution models should also assist in outbreak investigations; for example, ongoing research is examining the utility of the *E. coli* attribution models to understand sources and threat from different serotypes of STEC in the UK. Overall, the study underlines the need to optimize One Health surveillance programmes towards identification of interventions that can protect public health.

## Supplementary material

10.1099/mgen.0.001693 Uncited Supplementary Material 1.

10.1099/mgen.0.001693 Uncited Supplementary Material 2.

## Abbreviations

AMR: antimicrobial resistance
FSS: Food Standards Scotland
LGOCV: leave-one-group-out cross-validation
ML: machine learning
PATH-SAFE: PATHogen Surveillance in Agriculture, Food and the Environment
ST: sequence type
STEC: Shiga toxin-encoding *Escherichia coli*
UTI: urinary tract infection
WGS: whole-genome sequencing
